# Probiotic (*Enterococcus faecium*) induced responses of the hepatic proteome improves metabolic efficiency of broiler chickens (*Gallus gallus*)

**DOI:** 10.1186/s12864-016-2371-5

**Published:** 2016-02-01

**Authors:** Aijuan Zheng, Jianjie Luo, Kun Meng, Jianke Li, Wayne L. Bryden, Wenhuan Chang, Shu Zhang, L. X. N. Wang, Guohua Liu, Bin Yao

**Affiliations:** Key Laboratory of Feed Biotechnology of Ministry of Agriculture, Feed Research Institute, Chinese Academy of Agricultural Sciences, Beijing, 100081 People’s Republic of China; Key Laboratory of Pollinating Insect Biology of Ministry of Agriculture, Institute of Apicultural Research, Chinese Academy of Agricultural Sciences, Beijing, 100093 People’s Republic of China; School of Agriculture and Food Sciences, University of Queensland, Gatton, QLD 4343 Australia

**Keywords:** Broiler chicken (*Gallus gallus*), *Enterococcus faecium*, Liver, Proteome, Probiotics

## Abstract

**Background:**

The liver plays important roles in nutrient metabolism, detoxification and immunity. *Enterococcus faecium* (*E. faecium*) is a probiotic that has been shown to have positive effects on broiler production. However, its molecular effects on liver metabolism have not been characterized. This study aims to further identify the biological roles of *E. faecium* by characterizing the hepatic proteomic changes of broilers (*Gallus gallus*) fed *E. faecium* using two-dimensional fluorescence difference gel electrophoresis (2-D DIGE) and mass spectrometry (MS).

**Results:**

Thirty-three proteins (50 protein spots) involved in nutrient metabolism, immunity and the antioxidant system were shown to be differentially expressed in the liver of broilers fed *E. faecium* than from birds not fed the probiotic. The biological processes of sulphur amino acids, vitamin and cellular hormone metabolism, sulphur compound biosynthesis and protein tetramerization were enhanced in the liver of broilers fed *E. faecium*. However, proteins involved in calcium ion flux, cell redox homeostasis and platelet activation related to hepatic immune responses were down-regulated in broilers fed *E. faecium*. These results indicate that the supplementation of poultry feed with *E. faecium* may alter the partitioning of nutrients and promote optimal nutrient utilization.

**Conclusions:**

This study assists in unraveling the molecular effects of the dietary probiotic, *E. faecium*, in the liver of broiler chickens. It shows that the probiotic improves the metabolism of nutrients and decreases inflammatory responses. Our findings extend previous knowledge of the mechanism of dietary probiotic action and provide new findings for research and future probiotic development.

**Electronic supplementary material:**

The online version of this article (doi:10.1186/s12864-016-2371-5) contains supplementary material, which is available to authorized users.

## Background

Probiotics are live, non-pathogenic microorganisms that can regulate immune responses and suppress inflammation of intestinal cells [1]. In so doing, probiotics have positive effects on the gut microbiota [2]. Probiotics, along with plant extracts [3] and organic acids [4] are being increasingly used in chicken (broiler) meat production as alternatives to antibiotic growth promoters to improve broiler performance and enhance the sustainability of production. The application of antibiotics as growth promoters has been prohibited in the European Union because of increased antibiotic resistance [5] and in response to consumers demands for safe, and quality broiler meat products.

*Enterococcus faecium* is a facultative anaerobic lactic acid bacterium found in the intestinal microbiota of humans and animals [6]. As one of the direct-fed microorganism strains recognized by the Association of American Feed Control Officials, *E. faecium* is permitted as a probiotic supplement for broiler chicken diets [7, 8]. Its impact on broiler chickens include promotion of immune organ development [9], increased diversity of the gut microbial population [10], resistance to infection [11], changes of antioxidant status [7, 10], and increased intestinal absorptive surface area [10, 12, 13]. The latter two effects have also been demonstrated in our previous studies of the proteome responses of the intestinal mucosa of broilers fed *E. faecium* [9]. We have also shown that this probiotic induced proteome changes that improved broiler meat quality and yield [14].

The liver plays a central role in nutrient metabolism, including glycogen storage, protein and lipid synthesis, detoxification, and production of chemicals necessary for digestion [15, 16]. There are many reports that the intestinal microbiota and its products indirectly affect the liver [6, 7, 9–11, 13]. As a ‘first pass’ organ exposed to the highest concentration of nutrients and other substances in the portal system, the liver is most vulnerable to their effects and represents a target organ to assess the effects of probiotics. Undoubtedly, studying variations of the hepatic proteome following dietary probiotic supplementation will provide further insights into the mechanisms of probiotic action. Thus, the aim of this study was to undertake a molecular characterization of hepatic metabolism of broiler chickens fed the probiotic *E. faecium*

## Methods

The study described in this paper was conducted in the Feed Research Institute, Chinese Academy of Agricultural Sciences (CAAS), Beijing, China. The care and use of all birds in this experiment was approved by the Animal Care and Use Committee of the Feed Research Institute of CAAS.

### Materials and chemical reagents

Microcapsules of *E. faecium* CGMCC 2516 [17, 18] (viable count ≥1 × 10^10^ cfu/g; Challenge Biotechnology Ltd. Co., Beijing, China) were used in the present experiment. All reagents for two-dimensional fluorescence difference gel electrophoresis (2-D DIGE) were purchased from Bio-Rad (Hercules, CA), Roche (Mannheim, Germany), GE Healthcare (Uppsala, Sweden) and Sigma-Aldrich (St. Louis, MO). The reagents for LC-Chip-ESI-QTOF-MS were purchased from Bruker Daltonics (Billerica, MA), Roche and J. T. Baker (Phillipsburg, NJ). 2-D Quant Kit was purchased from GE Healthcare.

### Bird management and dietary treatments

Arbor Acres (AA) broilers were purchased from the Huadu Chicken Co. (Beijing, China). A total of 216, 1-day-old, male AA broiler chicks were randomly divided into two groups, control and treatment. Each group had 9 replicates (cage) and each replicate contained 12 birds. The distribution of cages was arranged to avoid any location effects on the poultry house. The chickens were reared in two stages, starter (0–21 days) and grower (22–42 days), and fed appropriate corn-soybean meal diets (Additional file 1: Table S1) containing 1.0 × 10^6^ cfu/g (starter diet) and 1.2 × 10^6^ cfu/g (grower diet) of *E. faecium* (treatment) or not (control) for 42 days. In accordance with the AA Broiler Management Guide (Aviagen Group 2009), all chicks were inoculated and subjected to a photoperiod of 23 h light on days 0–7 and 20 h thereafter. The room temperature was 33–35 °C for days 0–3 and gradually reduced to 20 °C by day 28. Relative humidity was maintained at 60–70 % during the first week and then at 50–60 % for the remainder of the study.

### Sample preparation

On day 42, chickens from each group were randomly selected, electrically stunned, and manually slaughtered within 5 min [19]. The livers were removed and washed with PBS (NaCl 8 g/L, Na_2_HPO_4_ 1.44 g/L, KH_2_PO_4_ 240 mg/L, and KCl 200 mg/L, pH 7.2) to remove any blood and contaminants on the surface and immediately stored in liquid nitrogen [20]. Hepatic protein extraction was performed as described previously with some modifications [14]. Liver samples (100 mg) were homogenized with liquid nitrogen and dissolved in 1 mL of PBS (pH 7.0) containing EDTA-free protease inhibitor cocktail tablets (Roche). The proteins insoluble in PBS were extracted by lysis buffer (9 M urea, 2 M thiourea, 4 % CHAPS, 2 EDTA-free protease inhibitor cocktail tablets, pH 8.5) and combined with the PBS soluble proteins. Trichloroacetic acid was added at a ratio of 1:9, followed by 10-min incubation at –20 °C. After centrifugation at 15,000 × *g* at 4 °C for 10 min, the pellet was washed with cold acetone, incubated and re-centrifuged as described above. The pellet was then air dried, suspended in lysis buffer at the ratio of 1 mg: 10 μL. The protein concentration of the supernatant was determined by the 2-D Quant Kit.

### 2-D DIGE and image analysis

The pH of the proteins was adjusted to 8.5 with 50 mM NaOH, and the concentration was adjusted to 5 mg/mL with lysis buffer. Equal amounts of proteins from the 6 samples of each control and treatment group were pooled together as the internal standard. The proteins (50 μg) were then labeled individually with 400 pmol of Cy3, Cy5 or Cy2 (specific for internal standard) on ice for 30 min in the dark and then quenched with 1 μL of 10 mM lysine on ice for another 10 min. 2-D DIGE was performed as described with some modifications [21]. To avoid erroneous conclusions due to individual variations, the same quantity of proteins from the liver of three chickens were pooled as a biological replicate, and three biological replicates were acquired for each group.

Three 2-D DIGE gels were independently carried out with the same internal standard sample. The Cy3- and Cy5-labeled proteins (50 μg) were combined, and then mixed with 50 μg of Cy2-labeled internal standard. An equal volume of 2 × sample buffer (9 M urea, 2 M thiourea, 4 % CHAPS, 130 mM DTT, and 1 % IPG buffer, pH 3.0–10.0; GE Healthcare) was then added to the sample, followed by the addition of rehydration buffer (8 M urea, 2 % CHAPS, 45 mM DTT, 0.5 % IPG buffer, and a trace amount of bromophenol blue, pH 3.0–10.0) to a total volume of 450 μL. Samples were applied to 24-cm pH 3.0–10.0 IPG strips (Bio-Rad), and isoelectric focusing was performed using the IPGphor IEF system (GE Healthcare). The isoelectric focusing program was set as follows: 50 V for 14 h, Grd 500 V for 30 min, Step 500 V for 1 h, Grd 1000 V for 30 min, Step 1000 V for 1 h, Grd 8000 V for 3 h, and step 8000 V for 30000 Vh. The IPG strips on the concentrator were equilibrated in buffer A (375 mM Tris-HCl [pH 8.8], 6 M urea, 29.3 % glycerol, 2 % SDS, 1 % DTT and a trace amount of bromophenol blue) for 15 min at room temperature and followed by equilibration with buffer B (375 mM Tris-HCl [pH 8.8], 6 M urea, 29.3 % glycerol, 2 % SDS, 2.5 % iodoacetamid and a trace amount of bromophenol blue) for another 15-min incubation at room temperature.

Homogeneous polyacrylamide gels (12 %) were precast with low fluorescence glass plates using an Ettan DALT six-gel caster, and IPG strips were placed on top of it. Strips were overlaid with 0.5 % Agarose-LE (Affymatrix, Santa Clara, CA) in 1 × running buffer containing bromphenol blue and were run for 14–16 h (2 W per gel, overnight) at 16 °C in an Ettan DALT six electrophoresis system (GE Healthcare). All electrophoresis procedures were performed in dim light or in the dark. After the run was completed, the 2-D DIGE gels were scanned in situ using a Typhoon 9410 Variable Mode Imager (GE Healthcare) and analyzed by the DeCyder Differential Analysis Software (version 7.0, GE Healthcare) according to the manufacturer’s instructions.

### Identification of protein spots of differential abundance by LC-Chip ESI-QTOF-MS

The identification of protein spots was carried out as described by Begna et al. with some modifications [22]. Interesting protein spots from the preparative gels were in-gel digested and identified by LC-Chip ESI-QTOF-MS (Q-TOF 6520, Agilent, Santa Clara, CA). The tandem mass spectra were retrieved using the Mass Hunter software (Version B.02.01, Agilent). Before the MS/MS data search, a peak-list was generated by the Mascot Distiller software (Version 3.2.1.0, Matrix Science, Boston, MA). The MS/MS data were searched against Mascot 2.2 (Matrix Science) applied to NCBInr (released March 2015) with the following parameters: carbamidomethylation (C) and oxidation (M) were the fixed and variable modifications, respectively; taxonomy, all entries; enzyme, trypsin/P; missed cleavages, 1; peptide tolerance, ± 20 ppm; and MS/MS tolerance, ± 0.02 Da. When the identified peptides were matched to multiple members of a protein family, or a protein appeared under the same name and accession number, the match was made in terms of the higher Mascot score, the putative function, and the differential patterns of the protein spots on the 2-D DIGE gels. Protein identifications were accepted if they established a probability greater than 95 % and contained at least two identified peptides having maximal peptide coverage. The relative abundances of differentially expressed proteins were normalized by the internal standard of pooled Cy2-labeled proteins.

### Bioinformatics analysis of differentially abundant proteins

The ClueGo software http://apps.cytoscape.org/apps/cluego with the Gene Ontology (GO) database (released March 2015) and Kyoto encyclopedia of genes and genomes (KEGG) database (released March, 2015) was used to classify identified proteins into specific functional terms and a pathway enrichment analysis. The gene ontology analysis based on biological process and enrichment analysis was performed by the right-side hyper-geometric statistic test and its probability value was corrected by the Bonferroni’s method [23]. While pathway enrichment analysis was performed by using the ClueGo software and the Gallus gallus database from KEGG database.

A protein interaction network of differential proteins was analyzed using the online database resource Search Tool (http://string-db.org/) for the Retrieval of Interacting Genes (STRING 9.05) [24]. The protein regulation networks and protein interaction maps are in the *Gallus gallus* molecular networks database. The network nodes are the proteins, and the edges represent the predicted functional associations. An edge may be drawn with up to seven differently colored lines—these lines representing the existence of seven types of evidence used in interaction prediction. The interactions between the imported proteins and all proteins stored in the database were then identified.

### Validation of proteins of differential abundance by qPCR

To further understand the relationship between proteins and their encoding genes, qPCR was run for proteins of differential hepatic abundance at the mRNA level. Specific primers for target genes of the identified proteins were designed using the primer BLAST of NCBI and nucleotide information in GenBank (Additional file 2: Table S2). Total RNA was prepared from the liver of control and treated groups using TRNzol-A+ (TIANGEN, Beijing, China). RNA quality and concentration were detected using spectrophotometer (Ultrospec 2100 pro, GE Healthcare) and agarose gel electrophoresis. cDNA synthesis with 5 μg of RNA was performed using the Fast Quant RT Kit (with gDNase) (TIANGEN). qPCR was conducted using the iCycler iQ5 system. The PCR was performed in a 20-μL reaction system containing 1 μL of cDNA, 0.5 μL of each primer (10 μM), 10 μL of Super Real PreMix (SYBR Green) (TIANGEN) and 8.2 μL of water. The fold-change was calculated using the IQTM5 software (Bio-Rad) with the 2 ^−ΔΔCt^ method [25]. All operation for qPCR was followed by the MIQE [26].

## Results

The broiler chickens were clinically normal throughout the experiment. As reported previously, dietary supplementation with *E. faecium* did not significantly increase growth rate or feed intake of the broilers. However, feed conversion efficiency was improved [9], along with meat quality and yield [14].

### Identification of proteins of differential abundance

**Fig. 1:**
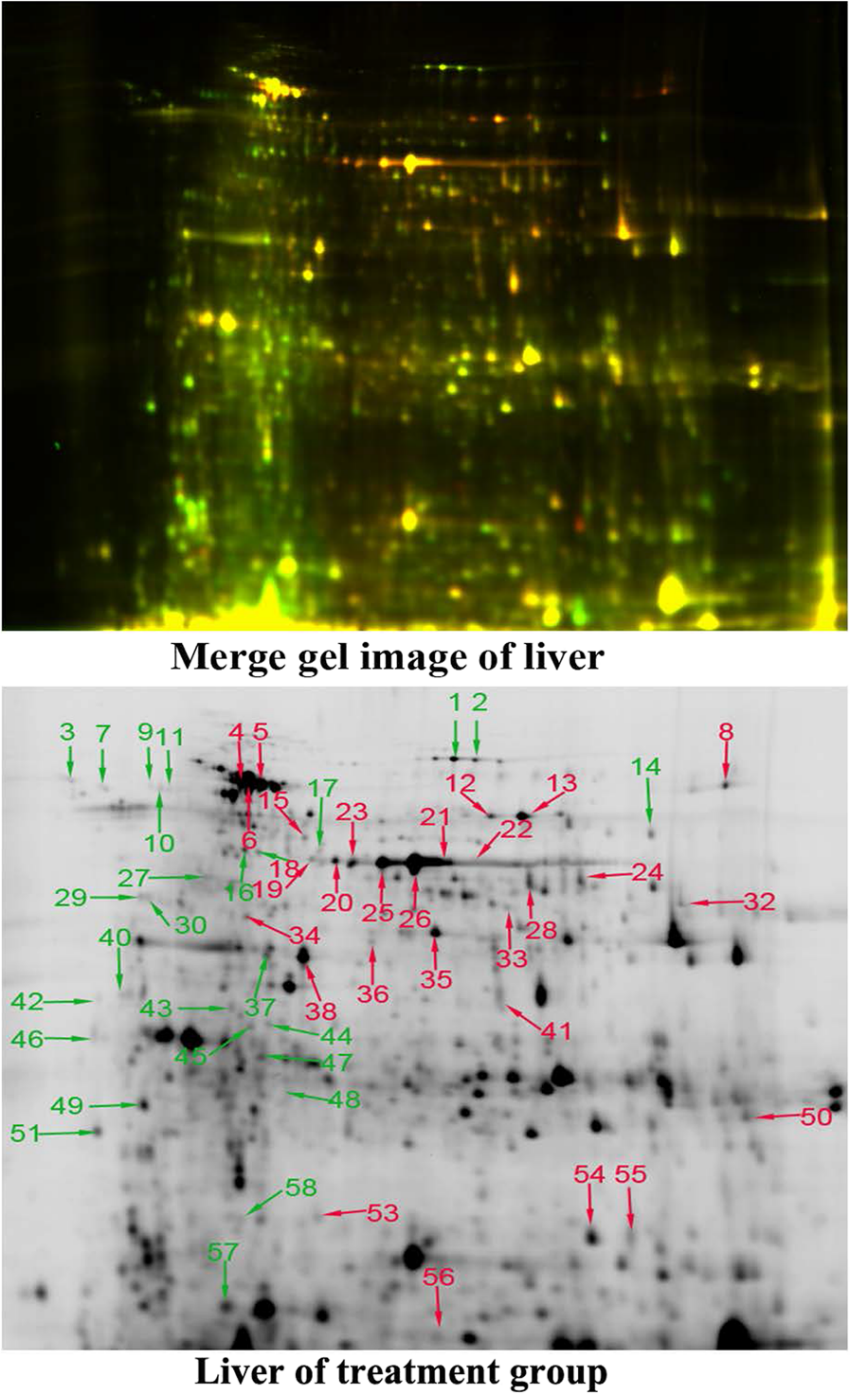
2D-DIGE hepatic protein profiles of broilers fed the probiotic *E. faecium* or not. Protein spots showing significant differences (1.4-fold, *p* < 0.05) were cut out and identified by LC-Chip-ESI-QTOF-MS. Proteins of differential abundance with known identities are number-labeled and marked red and green for up-regulation and down-regulation, respectively

A total of 213 protein spots were detected on 2-D DIGE gels of liver, with molecular weights and *p*I ranging from 10 to 100 kDa and 3.0 to 10.0, respectively (Fig. 1). There were 58 protein spots that displayed significantly different expression (1.4-fold, *p* < 0.05) between control and treatment groups. Fifty protein spots (86 %) were identified by MS (Table 1), with remainder unidentified, due to weak spectra.

**Table 1:** Differentially expressed proteins detected in the liver of AA broiler chickens fed the dietary probiotic *E. facecium* ^a^

Spot no.	Protein name	Accession no.	Symbol ID	*M*r (kDa)*/p*I	Sequence coverage (%)	Matched/searched	Mascot score	Av. ratio (treatment/control)	*p* value
Immune and antioxidant system									
1	Transferrin	gi|83754919	LTF	77.49/6.70	50	75/326	1282	−1.59	7.6E − 03
2	Transferrin	gi|83754919	LTF	77.49/6.70	50	75/326	1282	−1.60	1.4E − 02
3	PIT 54 protein	gi|46395491	PIT54	52.67/4.61	23	16/252	272	−1.61	8.5E − 04
4	Serum albumin precursor	gi|45383974	ALB	71.87/5.51	76	96/411	2211	2.10	1.2E − 02
5	Serum albumin precursor	gi|45383974	ALB	71.87/5.51	80	118/411	2390	1.73	2.0E − 02
6	Serum albumin precursor	gi|45383974	ALB	71.87/5.51	80	100/411	2671	2.39	3.4E − 03
7	PIT54 protein	gi|46395491	PIT 54	52.67/4.61	23	16/252	272	−1.67	6.7E − 05
14	Fibrinogen β chain	gi|399491	FGB	53.27/7.18	46	29/222	619	−1.56	3.2E − 03
15	Serum albumin precursor	gi|45383974	ALB	71.87/5.51	31	23/263	379	1.47	1.8E − 02
16	Fibrinogen gamma chain	gi|8569623	FGG	47.49/5.40	28	19/246	296	−1.57	1.5E − 02
18	Fibrinogen gamma chain	gi|8569623	FGG	47.49/5.40	26	12/191	232	−1.70	5.8E − 04
12	Catalase	gi|53127216	CAT	60.28/8.09	39	39/290	685	2.14	8.0E − 03
13	Catalase	gi|53127216	CAT	60.28/8.09	67	68/334	1104	2.39	1.4E − 03
34	Epoxide hydrolase 2	gi|75832164	EPHX2	63.72/5.89	19	18/201	308	1.43	4.2E − 02
40	HSP108	gi|63509	HSP90B1	91.45/4.81	4	8/241	86	−1.69	1.1E − 02
46	HSP108	gi|63509	HSP90B1	91.45/4.81	3	2/241	56	−1.48	1.8E − 02
49	Annexin A6	gi|50982399	ANXA6	75.58/5.57	8	4/244	71	−1.64	2.3E − 03
50	GlutathioneS-transferase 2	gi|2981970	GSTM2	25.92/7.00	63	18/217	546	1.48	3.9E − 03
51	HSP108	gi|63509	HSP90B1	91.45/4.81	13	16/209	402	−1.76	2.4E − 02
Carbohydrate metabolism and energy production									
20	α-Enolase	gi|46048768	ENO1	47.62/6.17	48	43/296	882	1.94	3.2E − 03
21	α-Enolase	gi|46048768	ENO1	47.62/6.17	38	17/227	378	1.78	4.8E − 03
23	α-Enolase	gi|46048768	ENO1	47.62/6.17	52	28/279	821	1.76	5.2E − 03
25	α-Enolase	gi|46048768	ENO1	47.62/6.17	41	39/291	880	1.77	1.3E − 02
26	α-Enolase	gi|46048768	ENO1	47.62/6.17	53	52/324	1088	1.80	6.6E − 03
27	Mitochondrial inner membrane protein	gi|57530041	IMMT	79.54/5.72	6	4/266	50	−1.74	5.4E − 03
36	α-Enolase	gi|46048768	ENO1	47.62/6.17	11	5/290	93	1.44	4.3E − 04
37	Phosphoglycolate phosphatase	gi|71894743	PGP	33.55/5.53	33	15/271	364	1.50	9.4E − 03
44	α-Enolase	gi|46048768	ENO1	47.62/6.17	8	4/296	62	−1.41	3.9E − 02
47	α-Enolase	gi|46048768	ENO1	47.62/6.17	23	11/240	313	−1.65	2.1E − 02
55	Alcohol dehydrogenase 6	gi|45384164	ADH6	40.89/7.85	16	8/231	130	3.38	2.2E − 03
56	Phosphoenolpyruvate carboxykinase [GTP], mitochondrial	gi|45382653	PCK2	71.72/8.16	11	9/196	134	1.65	7.8E − 03
58	Phosphoglycerate kinase	gi|45384486	PGK1	45.09/8.31	15	14/256	333	−1.45	1.5E − 02
Amino acid and protein metabolism									
17	Alanyl-tRNA synthetase, cytoplasmic	gi|57524852	AARS	102.00/5.68	11	14/189	272	−1.47	4.5E − 03
22	Homogentisate 1,2-dioxygenase	gi|50729534	HGD	50.12/6.35	34	17/279	397	1.79	3.6E − 03
24	Betaine-homocysteine S-methyltransferase 1	gi|50755288	BHMT	45.55/7.56	52	27/265	507	1.78	3.8E − 04
29	Protein disulfide-isomerase A4	gi|57530768	PDIA4	71.29/4.96	11	7/228	115	−1.62	2.8E − 03
32	Aspartate aminotransferase, cytoplasmic	gi|809192	GOT1	46.00/8.26	54	32/245	943	1.44	4.4E − 02
33	Cystathionase	gi|118094764	CTH	44.56/6.86	38	17/233	335	1.47	4.8E − 02
41	Betaine-homocysteine S-methyltransferase	gi|50755288	BHMT	45.55/7.56	32	18/259	316	1.49	1.4E − 02
42	Prolyl-4-hydroxylase	gi|63739	P4HB	55.17/4.66	13	7/250	146	−1.89	1.1E − 03
43	Protein disulfide-isomerase A3 precursor	gi|45383890	PDIA3	56.55/5.76	28	24/238	589	−1.42	4.4E − 03
45	Elongation factor 2	gi|45382453	EEF2	96.34/6.40	6	7/255	94	−1.40	2.1E − 03
Lipid and vitamin metabolism									
28	Retinal dehydrogenase 1	gi|45383031	ALDH1A1	56.40/7.49	16	14/229	111	1.41	3.7E − 02
35	3-oxo-5-β-steroid 4-dehydrogenase isoform 2	gi|118082901	AKR1D1	50.02/9.32	52	26/256	655	1.44	4.4E − 02
38	Regucalcin	gi|45382019	RGN	33.67/5.77	75	39/286	1279	1.41	2.7E − 03
48	Apolipoprotein A-I	gi|211159	APOA1	30.67/5.58	10	3/250	59	−1.42	3.6E − 02
57	Fatty acid-binding protein, liver	gi|45383728	FABP1	14.30/7.74	66	17/286	461	−1.90	2.6E − 03
Nucleotide metabolism									
8	Bifunctional purine biosynthesis protein PURH	gi|28373618	ATIC	67.05/8.54	31	30/230	364	2.46	1.7E − 03
53	Dihydropyrimidinase	gi|118087274	DPYS	69.50/6.42	4	2/265	70	1.51	4.5E − 02
54	Nucleoside diphosphate kinase	gi|2827446	NME4	17.54/7.11	65	27/277	492	1.43	6.8E − 03

^a^ Spot no. corresponds to the number of protein spots in Fig. 1. Protein name is given when proteins were identified by LC-Chip ESI-QTOF MS. Accession no. is the unique number given to mark the entry of a protein in the database NCBInr. Theoretical molecular weight (*Mr*) and isoelectric point (*p*I) of the identified proteins are retrieved from the protein database of NCBInr. Sequence coverage is the ratio of the number of amino acids in every peptide that matches with the mass spectrum divided by the total number of amino acids in the protein sequence. Matched peptide is the number of paring an experimental fragmentation spectrum to a theoretical segment of protein and searched peptide is the total searched peptide. Peptides were identified from the liver of AA broiler chickens based on Mascot scores (Additional file 3: Table S3) . Mascot scores are derived from ion scores as a non-probabilistic basis for ranking protein hits. Av. ratio and *p* value are calculated using DeCyder software version 7.0

### GO and KEGG function enrichment analysis

**Fig. 2:**
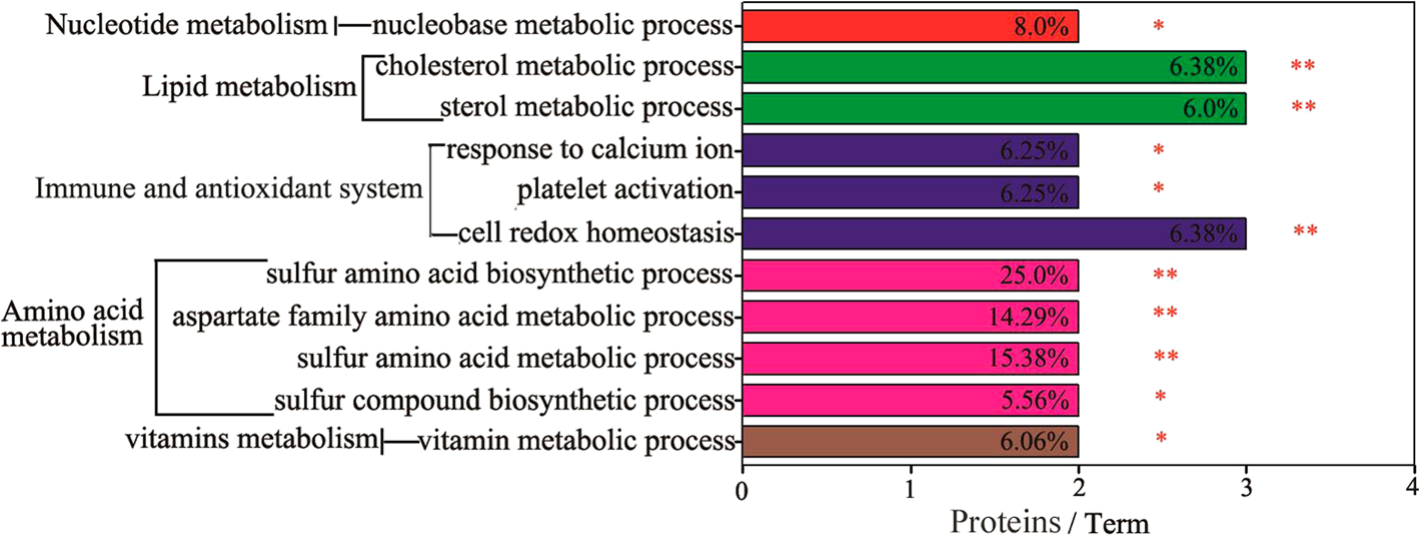
Functional enrichment analysis of the proteins of differential abundance in the livers of broilers fed the probiotic *E. faecium*. using ClueGO software. * and ** mean *p* < 0.05 and *p* < 0.01 levels of significance

The GO annotation was used to determine the biological events behind the data and to provide a primary overview of the hepatic proteome. Use of the ClueGo software allowed the functional enrichment analysis based on biological processes (Fig. 2). Five major functional groups were significantly enriched, i.e., amino acid metabolism, lipid metabolism, vitamin metabolism, nucleotide metabolism, and immunity and antioxidant system. With the sulphur amino acid biosynthetic process as the leading term (a term highly statistically significant or with the lowest *p*-value), proteins involved in amino acid metabolism contained BHMT (betaine-homocysteine S-methyltransferase 1, spots 24 and 41), CTH (cystathionase, spot 33), and GOT1 (aspartate aminotransferase, spot 32). Similarly, with cell redox homeostasis as the leading term, the immunity and antioxidant system functional group contained CAT (catalase, spots 12 and 13), FGB (fibrinogen β chain, spot 14) and FGG (fibrinogen gamma chain, spots 16 and 18). With the cholesterol metabolic process as the leading term, the lipid metabolism functional group contained AKR1D1 (3-oxo-5-β-steroid 4-dehydrogenase isoform 2, spot 35) and APOA1 (apolipoprotein A-I, spot 48). With the vitamin metabolic process as the leading term, the vitamin metabolism functional group contained ALDH1A1 (Retinal dehydrogenase 1, spot 28) and RGN (regucalcin, spot 38). The nucleotide metabolism functional group contained ATIC (bifunctional purine biosynthesis protein PURH, spot 8) and DPYS (dihydropyrimidinase, spot 53). The supplementation with *E. faecium* significantly up-regulated the sulphur amino acid biosynthetic and metabolic process, vitamin and cellular hormone metabolic process and protein tetramerization in the liver of broiler chickens. However, the biological process of glycerol ether and organic ether metabolism, cell redox homeostasis, platelet activation and response to calcium ion were down-regulated by dietary supplementation with *E. faecium.*

The KEGG pathway analysis showed that eight differentially expressed proteins were significantly enriched in the five pathways, which participate in multiple biological processes (Table 2). These pathways were mainly related to glycolysis/gluconeogenesis, retinol metabolism, and amino acid metabolism. Of them, the metabolism of glycine, serine, threonine, cysteine, methionine and tyrosine and the retinol metabolism pathway were enhanced according to the up-regulated proteins that were enriched (Tables 1 and 2).

**Table 2:** Enriched KEGG pathway-based sets of proteins of differential abundance in the liver of AA broiler chickens fed the probiotic *E. faecium* ^*a*^

Pathway name	Count	Protein	*p* value	*q* value
Glycolysis/Gluconeogenesis	3	ADH6, PGK1, ENO1	1.67E − 03	8.33E − 03
Glycine, serine and threonine metabolism	2	BHMT, CTH	1.29E − 02	6.46E − 02
Cysteine and methionine metabolism	3	BHMT, CTH, GOT1	3.58E − 04	1.79E − 03
Tyrosine metabolism	3	ADH6, GOT1, HGD	5.35E − 04	2.68E − 03
Retinol metabolism	2	ADH6, ALDH1A1	9.49E − 03	4.74E − 02

^a^ The number of count refers to the amount of proteins which are part of the extended network and appear as part of the pathway. *p* values are calculated according to a hypergeometric test, *q* values represent *p* values corrected for multiple testing using the false discovery rate method. Protein refers to the proteins involved in the corresponding pathway

### Protein and protein interaction analysis

**Fig. 3:**
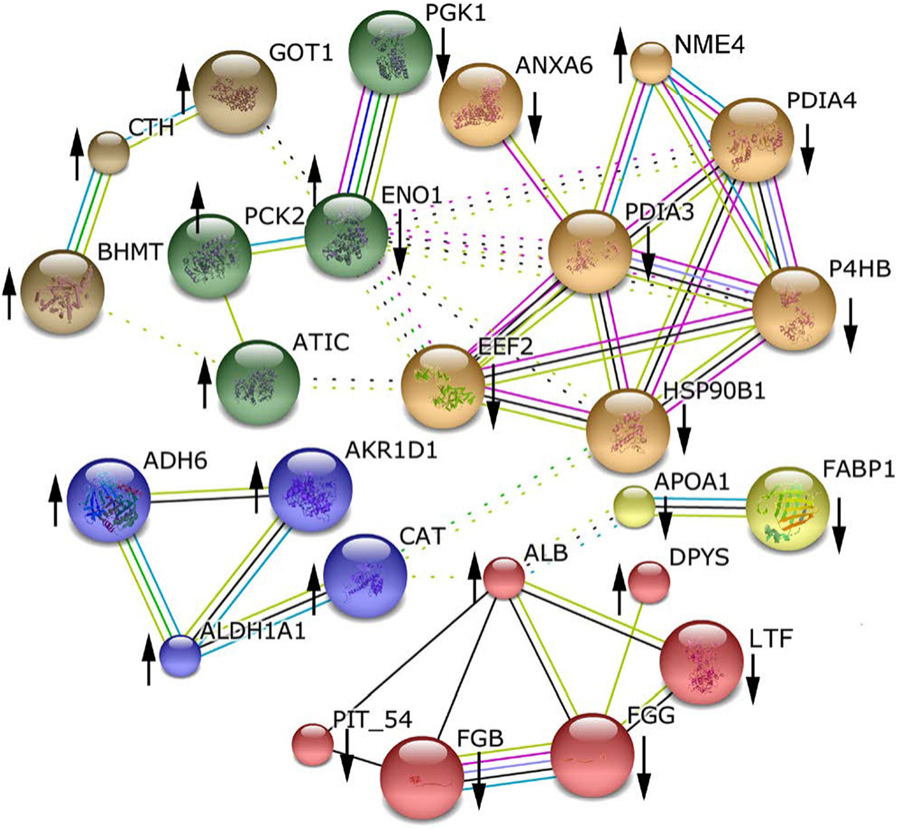
Biological interaction network of the proteins of differential abundance in the livers of broilers fed the probiotic *E. faecium*. Red lines indicate fusion evidence, green lines indicate neighborhood evidence, blue lines indicate co-occurrence evidence, purple lines indicate experimental evidence, yellow lines indicate text mining evidence light blue lines indicate database evidence, and black lines indicate co-expression evidence. ↑ and ↓ indicate up-regulated and down-regulated proteins in the livers of broilers fed the probiotic *E. faecium*, respectively

Of the 58 differentially expressed protein spots, twenty-six proteins were recognized as nodes with various relationships in the biological interaction networks (BIN) using the online tools of STRING 10.0 (Fig. 3). The node proteins were separated into six clusters, connected by twelve key node proteins (ENO1, EEF2, HSP90B1, PDIA3, PDIA4, P4HB, CAT, ALB, APOA1, ATIC, GOT1 and BHMT). The up-regulation of CTH, GOT1 and BHMT may be related to cysteine and methionine metabolism, while the down-regulation of HSP90B1 together with PDIA3, PDIA4, P4HB and EEF2 involves protein processing in the endoplasmic reticulum. The down-regulation of APOA1 and FABP1 may influence the uptake, transportation and deposition of fatty acids. The interaction of up-regulation of ALB and down-regulation of FGB, FGG and LTF probably plays a vital role in immunity and antioxidation. Other BIN proteins (PGK1, ENO1, PCK2 and ATIC) may function in carbohydrate metabolism. Of these, HSP90B1, ENO1, EEF2, and ALB are the most important hub proteins in the BIN system.

### Validation of proteins of differential abundance by qPCR

**Fig. 4:**
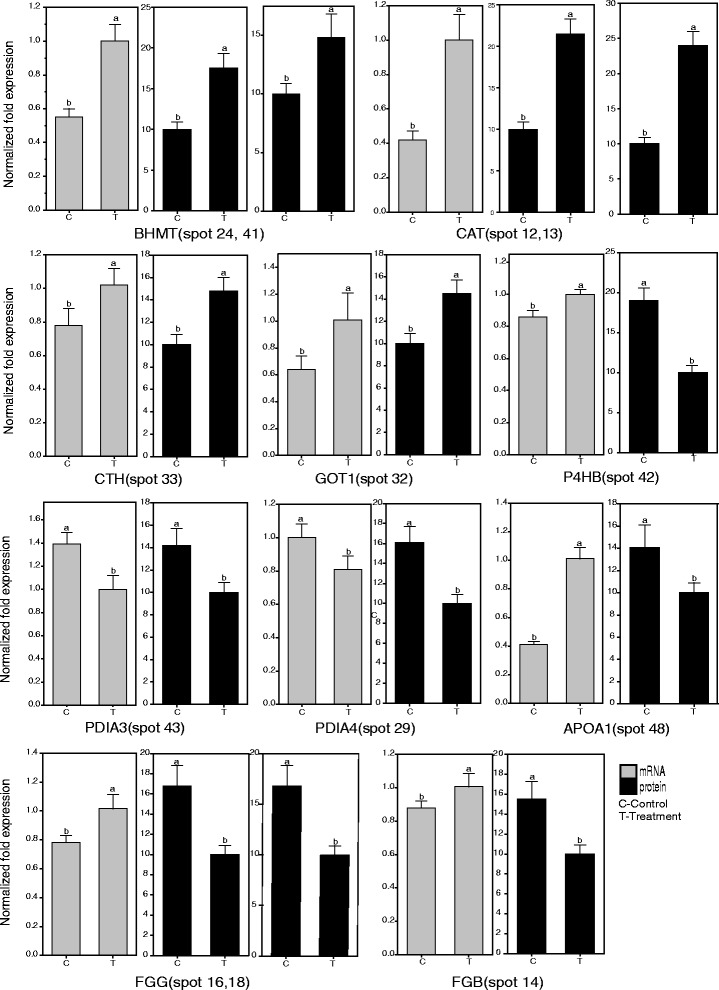
Validation using qPCR of ten proteins of differential abundance at the mRNA level in the livers of broilers fed the probiotic *E. faecium*. Samples were normalized with the reference genes 28S rRNA and *actin*. Gray and black histograms show the levels of mRNA and protein, respectively

Of the liver proteins with differential abundance, ten proteins that played an important role in nutrient metabolism (amino acid and lipid metabolism) and the immune system were selected to validate their expression at the level of mRNA (Fig. 4). The results showed that six of ten, CAT (spots 12 and 13), BHMT (spots 24 and 41), PDIA4 (spot 29), GOT1 (spot 32), CTH (spot 33), and PDIA3 (spot 43), were consistent with their mRNA expression levels. The similar expression pattern at the transcript level indicates a prospective opportunity for reverse genetic research through gene manipulation at different developmental stages of chickens. The other four genes, FGB (spot 14), FGG (spots 16 and 18), P4HB (spot 42) and APOA1 (spot 48), showed an inconsistent pattern between the mRNA and protein expression level.

## Discussion

The liver is the major metabolic organ in the body and it modulates the complexity of the processes of metabolism. Understanding the alterations that occur in hepatic metabolism following the administration of a probiotic to poultry, is an important aspect of delineating the mechanism of probiotic action and determining use strategies. The birds sampled in this study, used their feed more efficiently [9] and had improved carcass yield and quality [14].

The broiler chickens fed *E. faecium* had a significantly different hepatic protein expression profile than birds not exposed to the probiotic. Dietary supplementation *with E. faecium* significantly changed the immune and antioxidant system and the metabolism of carbohydrates, amino acids, nucleotides, vitamins, and lipids in the liver. In this regard, sulphur amino acids, vitamin and cellular hormone metabolism, sulphur compound biosynthesis and protein tetramerization were enhanced following feeding with *E. faecium*. In contrast, proteins involved in calcium ion flux, cell redox homeostasis and platelet activation were down-regulated. These results indicate that dietary supplementation with *E. faecium* may alter the partitioning of nutrients, thus facilitating optimal nutrient utilization.

The liver plays an important immunological role [27]. When inflammation occurs, the acute phase response often appears in the liver [28]. This response is a natural systemic defense to help protect the body against infections, neoplasm, stress, immune disorders, trauma or parasites [29]. Acute phase proteins (APPs), synthesized in the liver during the acute phase response [30], can be divided as positive or negative based on increase or decline of their concentrations during inflammatory responses [31, 32]. Positive APPs in avian species include ceruloplasmin, α1-acid glycoprotein, amyloid A, transferrin (LTF), mannan-binding protein, haptoglobin, hemopexin, fibrinogen and fibronectin [33], while albumin (ALB) was reported to a negative APP in poultry [34]. In this study, down-regulation of positive APPs including LTF, FGB and FGG and up-regulation of negative APP, such as ALB, suggest a possible relationship between supplementation with *E. faecium* and decreased inflammation. This result is consistent with that observed in the intestinal mucosa and immune organ indexes of these broilers [9]. Moreover, HSP90B1 is an essential immune chaperone, regulating innate and adaptive immunity [35]. Therefore, down-regulation of HSP90B1 (spots 40, 46, and 51) and differential expression of APPs in the liver of treated broilers suggests that supplementation with *E. faecium* may improve broiler health as the concentration of APPs may be useful for monitoring domestic fowl health [36]. Under normal physiological conditions, reduced inflammation will result in more efficience in animal production [37]. Chickens with high concentrations of positive APPs had growth depression and decreased meat quality because of repartitioning of nutrients away from muscle protein deposition and growth to APPs synthesis, in response to inflammation [38, 39]. In our study, the higher efficiency of production in the broilers fed with *E. faecium* may be due to the redistribution of nutrients away from the immune response resulting in increased availability of nutrients for growth and development.

Oxidative stress can induce cellular damage and cause disruption of normal cell signaling by over reactive oxygen species [40]. Antioxidant enzymes can protect cells from oxidative stress damage [41, 42]. High-level expression of antioxidant enzymes, i.e. CAT (spots 12 and 13), epoxide hydrolase 2 (EPHX2, spot 34), and glutathione S-transferase 2 (GSTM2, spot 50), suggests that the broilers fed *E. faecium* have synthesized more antioxidants to protect their livers from oxidative damage.

Dietary carbohydrates provide more than half the energy requirement, demonstrating the importance of hepatic carbohydrate metabolism to broiler chicken health and production. Proteins related to carbohydrate metabolism that were up-regulated in the liver of chickens fed *E. faecium*, included ENO1 (spots 20, 21, 23, 25, 26, and 36), phosphoglycolate phosphatase (PGP, spot 37), ADH6 (spot 55), and phosphoenolpyruvate carboxykinase (PCK2, spot 56). These proteins are involved in gluconeogenesis and glycolysis. However, phosphoglycerate kinase which is also involved in glycolysis (PGK1, spot 56) was down-regulated. The results suggest that supplementation of *E. faecium* significantly changed the carbohydrate metabolic pathways. ENO1 is a multifunctional protein that plays roles in glycolysis and carbohydrate degradation. Human ENO1 has 32 post-translational modification sites for phosphorylation, acetylation and ubiquitination (http://www.uniprot.org/uniprot/P06733#publications), and its functional roles depend on subcellular localization, post-translational modifications and the concentrations of modified protein [43]. In this study, eight differential protein spots of ENO1 were identified in the liver of broilers as reported in *Plasmodium yoelii* [44], including six up-regulated and two down-regulated. It suggests that supplementation of *E. faecium* may change the abundance and diversity of modified ENO1, and its physiological functions as a consequence. However, how and which ENO1 isoforms play the physiological roles requires further study.

Proteins, accounting for about 18 percent of the body weight of animals, are vital constituents of all cells and play important roles in many biological activities. In this study, the proteins related to amino acid and protein metabolism were identified to differentially express, such as alanyl-tRNA synthetase (AARS, spot 17); PDIA4 (spot 29), P4HB (spot 42), PDIA3 (spot 43) and EEF2 (spot 45). These proteins are principally involved in the synthesis of proteins [45], rearrangement of intrachain and interchain disulphide bonds [46], and polypeptide chain elongation [47].

Lipids play physiologically important roles in regulatory metabolism, metabolic energy production and as constituents of cell membranes [48]. APOA1 (spot 48) constitutes the high density lipoprotein complex with lipids in the plasma, and transports lipids from tissue to liver for excretion. FABP1 (spot 57) primarily binds long-chain fatty acids and hydrophobic ligands [49] and is associated with fatty acid uptake [50]. The down-regulation of APOA1 and FABP1 in the liver may be related to a decline in abdominal fat percentage which was observed in these birds [14]. Vitamins play a role as metabolic catalysts in the form of coenzymes. The differential proteins involved in metabolism of vitamins and nucleotides were identified in the liver of broilers fed *E. faecium*. RGN plays major roles in L-ascorbic acid biosynthesis and regulates hepatic cell functions [51]. Up-regulated RGN in the liver of broilers fed *E. faecium* may be linked to enhanced vitamin C synthesis and increased broiler immunity.

Proteins serve as fundamental elements in the living cell but do not exist independently [52]. KEGG pathway enrichment analysis and BIN analysis of differentially expressed proteins is the best way to perform functional analysis [53, 54]. Significantly enriched biological pathways of glycolysis/gluconeogenesis, retinol metabolism, and amino acid metabolism were observed in this study, indicating their central roles in hepatic metabolic enhancement. The proteins included in the BIN were mainly involved in the immune and antioxidant system and metabolism of carbohydrates, amino acids, proteins, nucleotides, lipids and vitamins. The complex network would appear to indicate that supplementation of broilers with *E. faecium* is related to the improved cysteine and methionine metabolism, reduced lipid deposition, immuno-inflammatory responsiveness, and enhanced protein processing in hepatic endoplasmic reticulum. Thus, the important hub proteins in the interaction network, HSP90B1, FGG, FGB and APOA1 play the vital role in improving immune function and metabolism of the liver in broilers fed with *E. faecium*. Some of the key node proteins that were highly linked in the BIN were validated at a gene level. Proteins BHMT, CAT, CTH, GOT1, PDIA3 and PDIA4 with abundances corresponding to mRNA levels may represent potential targets for genetic manipulation. However, possible reasons for these inconsistent results might include: 1) delayed outcomes of some biological processes, such as transcription or translation initiation, elongation efficiency, mRNA stability, or splicing [55–57]; 2) post-translational modifications, e.g. phosphorylation, or proteolysis of core components of the translation machinery [58–60]; 3) time delays between responses at the mRNA and protein levels; and 4) different degradation rates of proteins and mRNAs [61, 62]. Manipulation of these genes may require further experimental information.

## Conclusion

This study assists in the unraveling of the molecular effects of dietary probiotics in the livers of broiler chickens by using proteomics technology. Fifty differentially expressed proteins were identified in the treated broilers, and most of them are related to the metabolism of glycine, serine, threonine, cysteine, methionine and tyrosine and inflammatory responses. These results suggest that dietary supplementation of *E. faecium* may alter the partitioning of nutrients in the body and facilitate optimal utilization of nutrients by broilers. This study extends our previous knowledge of the mechanism of dietary probiotic action and provides new findings for research and future probiotic development.

## Additional files

Additional file 1: Table S1.
**The composition of broiler chickens starter and grower diets (g/kg).** (DOC 43 kb) Additional file 2: Table S2.
**The primer sequences used for qPCR analysis of the differentially expressed proteins of the liver of AA broiler chickens.** (DOC 37 kb) Additional file 3: Table S3.
**Peptides identified from the liver of AA broiler chickens based on Mascot scores.** (DOC 103 kb)

## Abbreviations

2-D DIGE: two-dimensional fluorescence difference gel electrophoresis
AARS: Alanyl-tRNA synthetase
ADH6: Alcohol dehydrogenase 6
AKR1D1: 3-oxo-5-β-steroid 4-dehydrogenase isoform 2
ALB: serum albumin precursor
ALDH1A1: retinal dehydrogenase 1
APOA1: apolipoprotein A-I
APPs: acute phase proteins
ATIC: bifunctional purine biosynthesis protein PURH
BHMT: betaine-homocysteine S-methyltransferase 1
BIN: biological interaction networks
CAAS: Chinese Academy of Agricultural Sciences
CAT: catalase
CTH: cystathionase
DPYS: dihydropyrimidinase
E. faecium: enterococcus faecium
EEF2: elongation factor 2
ENO1: α-Enolase
EPHX2: epoxide hydrolase 2
FABP1: Fatty acid-binding protein liver
FGB: fibrinogen β chain
FGG: fibrinogen gamma chain
GO: Gene Ontology
GOT1: aspartate aminotransferase cytoplasmic
GSTM2: glutathione S-transferase 2
HGD: homogentisate 1,2-dioxygenase
HSP90B1: HSP108
KEGG: Kyoto encyclopedia of genes and genomes database
LTF: transferrin
MS: mass spectrometry
P4HB: prolyl-4-hydroxylase
PCK2: phosphoenolpyruvate carboxykinase
PDIA3: protein disulfide-isomerase A3 precursor
PDIA4: protein disulfide-isomerase A4
PGK1: phosphoglycerate kinase
PGP: phosphoglycolate phosphatase
PIT 54: PIT 54 protein
RGN: regucalcin
STRING: Search Tool for the Retrieval of Interacting Genes
