# Supplementary material for: Probiotic (Enterococcus faecium) induced responses of the hepatic proteome improves metabolic efficiency of broiler chickens (Gallus gallus)
Source: BMC Genomics. 2016 Feb 1;17:89. doi: 10.1186/s12864-016-2371-5 (PMC4736614; doi:10.1186/s12864-016-2371-5)
Supplement: Additional file 2: Table S2. — The primer sequences used for qPCR analysis of the differentially expressed proteins of the liver of AA broiler chickens. (DOC 37 kb) [file 12864_2016_2371_MOESM2_ESM.doc]

**Table S2. Peptides identified from the liver of AA broiler chickens based on Mascot scores ^a^.**

| Spot  no. ^a^ | Protein name | Species | Mascot score | Peptides |
| --- | --- | --- | --- | --- |
| 1 | Ovotransferrin | *Gallus gallus* | 1282 | **APPKSVIRWCTISSPEEKK**  **ATYLDCIKAIANNEADAISLDGGQVFEAGLAPYK**  **KGTEFTVNDLQGKTSCHTGLGRSAGWNIPIGTLIHRGAIEWEGIESGSVEQAVAKFFSASCVPGATIEQK**  **NAPYSGYSGAFHCLK**  **HTTVNENAPDQK**  **QPVDNYKTCNWARVAAHAVVAR**  **DSAIMLKR**  **KDQLTPSPR**  **IQWCAVGKDEK**  **TDERPASYFAVAVARKDSNVNWNNLKGKKSCHTAVGR**  **TGTCNFDEYFSEGCAPGSPPNSRLCQLCQGSGGIPPEKCVASSHEKYFGYTGALR**  **NKADWAKNLQMDDFELLCTDGRRANVMDYRECNLAEVPTHAVVVRPEK**  **RFGVNGSEKSK**  **CLFKVR**  **TCNPSDILQMCSFLEGK** |
| 2 | Ovotransferrin | *G. gallus* | 1282 | **APPKSVIRWCTISSPEEK**  **KGTEFTVNDLQGKTSCHTGLGR**  **GAIEWEGIESGSVEQAVAKFFSASCVPGATIEQK**  **VAAHAVVAR**  **KDQLTPSPR**  **IQWCAVGKDEK**  **KDSNVNWNNLKGK**  **SCHTAVGR**  **TGTCNFDEYFSEGCAPGSPPNSRLCQLCQGSGGIPPEK**  **NLQMDDFELLCTDGR**  **DLLERQEK**  **FGVNGSEK** |
| 3 | PIT54 protein | *G. gallus* | 272 | **LVGGPNR**  **QLGCGDAVLAPIAAK**  **QLGCGTALSALPESK**  **LVDGPNQCAGRVEVFHENRWGSVCDDNWDMK**  **QVGCGSPLSALGSARYGRGPDVIWLDDVNCEGTEESIFDCK** |
| 4 | Serum albumin precursor | *G. gallus* | 2211 | **DAEHKSEIAHRYNDLKEETFKAVAMITFAQYLQR**  **LVKDVVDLAQKCVANEDAPECSKPLPSIILDEICQVEKLRDSYGAMADCCSKADPERNECFLSFKVSQPDFVQPYQRPASDVICQEYQDNRVSFLGHFIYSVAR**  **ESDVGACLDTKEIVMR**  **QQYFCGILKQFGDRVFQARQLIYLSQKYPKAPFSEVSKFVHDSIGVHK**  **MMSNLCSQQDVFSGK**  **DCCEKPIVERSQCIMEAEFDEKPADLPSLVEKYIEDKEVCKSFEAGHDAFMAEFVYEYSRRHPEFSIQLIMRIAKGYESLLEKCCKTDNPAECYANAQEQLNQHIKETQDVVKTNCDLLHDHGEADFLK**  **MPQVPTDLLLETGKKMTTIGTK**  **MACSEGYLSIVIHDTCR**  **RPCFTAMGVDTKYVPPPFNPDMFSFDEKLCSAPAEEREVGQMKLLINLIKR**  **TIADGFTAMVDKCCKQSDINTCFGEEGANLIVQSR** |
| 5 | Serum albumin precursor | *G. gallus* | 2390 | **DAEHKSEIAHRYNDLKEETFKAVAMITFAQYLQR**  **LVKDVVDLAQKCVANEDAPECSKPLPSIILDEICQVEKLRDSYGAMADCCSKADPERNECFLSFKVSQPDFVQPYQRPASDVICQEYQDNRVSFLGHFIYSVAR**  **ESDVGACLDTKEIVMR**  **QQYFCGILKQFGDRVFQARQLIYLSQKYPKAPFSEVSKFVHDSIGVHKECCEGDMVECMDDMARMMSNLCSQQDVFSGK**  **DCCEKPIVERSQCIMEAEFDEKPADLPSLVEKYIEDKEVCKSFEAGHDAFMAEFVYEYSRRHPEFSIQLIMRIAKGYESLLEKCCKTDNPAECYANAQEQLNQHIKETQDVVKTNCDLLHDHGEADFLK**  **MPQVPTDLLLETGKKMTTIGTK**  **MACSEGYLSIVIHDTCR**  **RPCFTAMGVDTKYVPPPFNPDMFSFDEKLCSAPAEEREVGQMKLLINLIKRKPQMTEEQIKTIADGFTAMVDKCCKQSDINTCFGEEGANLIVQSR** |
| 6 | Serum albumin precursor | *G. gallus* | 2671 | **DAEHKSEIAHRYNDLKEETFKAVAMITFAQYLQR**  **LVKDVVDLAQKCVANEDAPECSKPLPSIILDEICQVEKLRDSYGAMADCCSKADPERNECFLSFKVSQPDFVQPYQRPASDVICQEYQDNRVSFLGHFIYSVAR**  **ESDVGACLDTKEIVMR**  **QQYFCGILKQFGDRVFQARQLIYLSQKYPKAPFSEVSKFVHDSIGVHKECCEGDMVECMDDMARMMSNLCSQQDVFSGK**  **DCCEKPIVERSQCIMEAEFDEKPADLPSLVEKYIEDKEVCKSFEAGHDAFMAEFVYEYSRRHPEFSIQLIMRIAKGYESLLEK**  **TDNPAECYANAQEQLNQHIKETQDVVKTNCDLLHDHGEADFLK**  **MPQVPTDLLLETGKKMTTIGTK**  **MACSEGYLSIVIHDTCR**  **RPCFTAMGVDTKYVPPPFNPDMFSFDEKLCSAPAEEREVGQMKLLINLIKRKPQMTEEQIKTIADGFTAMVDKCCKQSDINTCFGEEGANLIVQSR** |
| 7 | PIT54 protein | *G. gallus* | 234 | **QLGCGTALSSPKK**  **LVGGPNR**  **QLGCGDAVLAPIAAK**  **QLGCGTALSALPESK**  **LVDGPNQCAGRVEVFHENR**  **QVGCGSPLSALGSAR** |
| 8 | Bifunctional purine biosynthesis protein purh | *G. gallus* | 364 | **DVSDLTGFPEMLGGR**  **TLHPAVHAGILAR**  **VVVCNLYPFVKTVSSPGVTVPEAVEKIDIGGVALLR**  **VTVVCDPADYSSVAK**  **GVSQLPLRYGMNPHQSPAQLYTTRPK**  **TLTPLASAYAR**  **MSSFGDFIALSDICDVPTAK**  **EVSDGVVAPGYEEEALKILSK**  **NNAVIDRSLFK**  **DGQVIGIGAGQQSR**  **LAGDKANSWWLR** |
| 12 | CAT catalase | *G. gallus* | 685 | **DVASEQLKR**  **LNIMTVGPRGPLLVQDVVFTDEMAHFDRER**  **GAGAFGYFEVTHDITK**  **AKVFEHIGKR**  **FSTVAGESGSADTVRDPR**  **FYTEEGNWDLVGNNTPIFFIRDAMLFPSFIHSQK**  **GIPDGFR**  **LVNASGGAVYCK**  **NLSVEEAARLASTDPDYGIR**  **LFAYPDTHR**  **LGPNYLQIPVNCPYR**  **MSVSGDVQR**  **NIADHLK**  **NFTDVHPDYGAR** |
| 13 | CAT catalase | *G. gallus* | 1104 | **DVASEQLKR**  **GSQKPDALTTGAGNPIGDKLNIMTVGPRGPLLVQDVVFTDEMAHFDRER**  **GAGAFGYFEVTHDITK**  **VFEHIGKR**  **FSTVAGESGSADTVRDPR**  **FYTEEGNWDLVGNNTPIFFIRDAMLFPSFIHSQKR**  **GIPDGFRHMNGYGSHTFKLVNASGGAVYCKFHVKTDQGIKNLSVEEAARLASTDPDYGIR**  **IVLNRNPVNYFTEVEQMAYDPSNMPPGIEPSPDK**  **LFAYPDTHR**  **LGPNYLQIPVNCPYR**  **DGPMCVSDNQGGAPNYYPNSFTGPEDQPVLKESRMSVSGDVQRFSSANEDNVSQVRDFYLK**  **NIADHLKDAQLFIQK**  **AVKNFTDVHPDYGARIQALLDKYNAEAGK** |
| 14 | Fibrinogen beta chain | *G. gallus* | 619 | **GPIIYPDAGGCK**  **TVKPVLR**  **FSDTSTTMYQYVNMIDNKLVK**  **AVIDSLHKK**  **LENAIATQTDYCRSPCVASCNIPVVSGRECEDIYRKGGETSEMYIIQPDPFTTPYRVYCDMETDNGGWTLIQNRQDGSVNFGRAWDEYKR**  **YCDTPGEYWLGNDKISQLTK**  **DNDGWLTTDPR**  **EDGGGWWYNR**  **YYWGGTYSWDMAKHGTDDGIVWMNWK**  **IKPYFPD** |
| 15 | Serum albumin precursor | *G. gallus* | 379 | **ESDVGACLDTK**  **QQYFCGILK**  **QLIYLSQKYPKAPFSEVSKFVHDSIGVHK**  **MMSNLCSQQDVFSGK**  **SQCIMEAEFDEKPADLPSLVEKYIEDKEVCK**  **TNCDLLHDHGEADFLK**  **RPCFTAMGVDTKYVPPPFNPDMFSFDEKLCSAPAEEREVGQMK**  **KPQMTEEQIKTIADGFTAMVDK**  **QSDINTCFGEEGANLIVQSR** |
| 16 | Fibrinogen gamma chain | *G. gallus* | 296 | **ENCCILDER**  **QTLPQSIEQLTQK**  **SGLYFIKPQK**  **LDGSEDFRR**  **IELEDWSGKKGTADYAVFKVGTEEDKYR**  **DTGTNSYDNGIIWATWRDRWYSMK**  **IIPFNRLSIDGQQHSGGLKQVGDS** |
| 17 | Alanyl-tRNA synthetase, cytoplasmic | *G. gallus* | 272 | **GLEVTDDSPKYGYTSDPSGTYDFGSLVATVK**  **FVEEVSTGQECGIVLDR**  **VGDQVHLSIDETR**  **SVLGEADQR**  **AVFDETYPDPVR**  **RIVAVTGAEARK**  **VMDDLDRASK** |
| 18 | Fibrinogen gamma chain | *G. gallus* | 232 | **ENCCILDER**  **QTLPQSIEQLTQK**  **SGLYFIKPQK**  **RLDGSEDFRRNWVQYK**  **IELEDWSGKKGTADYAVFK**  **DTGTNSYDNGIIWATWRDRWYSMK**  **IIPFNRLSIDGQQHSGGLK** |
| 20 | α-Enolase | *G. gallus* | 882 | **GNPTVEVDLYTNK**  **AAVPSGASTGIYEALELR**  **AVEHVNKTIAPALISKNVNVVEQEKIDKLMLEMDGTENK**  **GVPLYR**  **IGAEVYHNLK**  **DATNVGDEGGFAPNILENKEALELLK**  **VVIGMDVAASEFYRDGKYDLDFK**  **NYPVVSIEDPFDQDDWAAWKK**  **SCNCLLLKVNQIGSVTESLQACKLAQSNGWGVMVSHR**  **YNQLLRIEEELGSKAR** |
| 21 | α-Enolase | *G. gallus* | 378 | **GNPTVEVDLYTNK**  **AAVPSGASTGIYEALELR**  **YLGKGVSK**  **TIAPALISKNVNVVEQEKIDKLMLEMDGTENK**  **GVPLYR**  **IGAEVYHNLK**  **DATNVGDEGGFAPNILENKEALELLK**  **DGKYDLDFK**  **SCNCLLLKVNQIGSVTESLQACKLAQSNGWGVMVSHR**  **IEEELGSKAR** |
| 22 | Homogentisate 1,2-dioxygenase | *G. gallus* | 397 | **YMSGFGNEHASEDPR**  **WKPFEIPKASQNKLDFVSGLHTLCGAGEPR**  **LLITTEFGKMLVEPNEICVIQQGMR**  **DFLVPVAWYEDRKIPGGYTVISK**  **STRPGVAIADFVIFPPRWGVANNTFRPPYYHRNCMSEFMGLIK**  **CWEPLKSHFNPNCK** |
| 23 | α-Enolase | *G. gallus* | 821 | **GNPTVEVDLYTNKAAVPSGASTGIYEALELR**  **AVEHVNKTIAPALISKNVNVVEQEKIDKLMLEMDGTENKSKFGANAILGVSLAVCK**  **GVPLYR**  **IGAEVYHNLK**  **DATNVGDEGGFAPNILENK**  **AGYSDKVVIGMDVAASEFYRDGKYDLDFK**  **SCNCLLLKVNQIGSVTESLQACKLAQSNGWGVMVSHRSGETEDTFIADLVVGLCTGQIK**  **YNQLLRIEEELGSKAR** |
| 24 | Betaine-homocysteine S-methyltransferase 1 | *G. gallus* | 507 | **RGILER**  **AGPWTPEATVEHPEAVR**  **AGSNVLQTFTFYASEDKLENR**  **VNEAACDIAR**  **KQLDIFMKK**  **AGASIVGVNCHFDPDTVLETVKLMKEGLQAAK**  **AHLMSQPLAFHTPDCGKQGFIDLPEFPFGLEPR**  **KAYDLGIRYIGGCCGFEPYHVRAIAEELAPERGFLPEASEK**  **EYWENLKPASGRPYCPSMSKPDGWGVTK**  **EATTEQQLKELFQK** |
| 25 | α-Enolase | *G. gallus* | 880 | **GNPTVEVDLYTNKGLFRAAVPSGASTGIYEALELR**  **YLGKGVSKAVEHVNKTIAPALISKNVNVVEQEKIDKLMLEMDGTENKSK**  **GVPLYR**  **IGAEVYHNLK**  **AGYSDKVVIGMDVAASEFYRDGKYDLDFK**  **SCNCLLLKVNQIGSVTESLQACKLAQSNGWGVMVSHR**  **YNQLLRIEEELGSK** |
| 26 | α-Enolase | *G. gallus* | 1088 | **GNPTVEVDLYTNKGLFRAAVPSGASTGIYEALELRDNDK**  **GVSKAVEHVNKTIAPALISKNVNVVEQEKIDKLMLEMDGTENKSK**  **GVPLYR**  **EAMRIGAEVYHNLKNVIKEKYGKDATNVGDEGGFAPNILENK**  **AGYSDKVVIGMDVAASEFYRDGKYDLDFK**  **FTASVGIQVVGDDLTVTNPK**  **SCNCLLLKVNQIGSVTESLQACKLAQSNGWGVMVSHR**  **YNQLLRIEEELGSK** |
| 27 | Mitochondrial inner membrane protein | *G. gallus* | 50 | **AVDEAADVLLK**  **IDQLNKELAEQR**  **EQQHIELALEK**  **KALEAAVAK** |
| 28 | Retinal dehydrogenase 1 | *G. gallus* | 111 | **KFEVFNPANEEKICEVAEGDKADIDK**  **KAFELGSPWR**  **LADLVERDR**  **TVPMDGNFFTFTR**  **IFVEEPIYDEFVRR**  **ILDLIESGKK** |
| 29 | Protein disulfide-isomerase A4 | *G. gallus* | 115 | **QFAPEYEK**  **TLKENDPPIPVAKIDATAATALASR**  **RTPPIPLAKVDATAETELAKKFDVTGYPTLK**  **GKPYDYSGPR** |
| 32 | Aspartate aminotransferase | *G. gallus* | 943 | **APPVAVFKLTADFREDGDSRKVNLGVGAYRTDEGQPWVLPVVR**  **IALGDDSPAIAQKRVGSVQGLGGTGALRIGAEFLRR**  **TYRYWDAAK**  **QIAAVMKR**  **CLFPFFDSAYQGFASGSLDK**  **YFVSEGFELFCAQSFSKNFGLYNERVGNLSVVGKDEDNVQR**  **TTWSNPPSQGARIVATTLTSPQLFAEWKDNVK**  **QVEYMIKEKHIYLMASGRINMCGLTTK**  **SIHEAVTKIQ** |
| 33 | Cystathionase | *G. gallus* | 335 | **SGALVPPVSLSTTFKQQAPGEHAGYDYSR**  **AGDTIICMDDVYGGTNRYFQQIAKK**  **CLEAAITPETKLVWIETPTNPTLKVIDIQGCADVIHK**  **MNQHFR**  **VIYPGLPSHPQHELAKR**  **HATIFLK**  **EALGISDTLIRLSVGLEDEEDLLEDLDQALK** |
| 34 | Epoxide hydrolase 2 | *G. gallus* | 308 | **YQIPALADAGFRVIALEMK**  **AVASLNTPYRPADPTVDIVETMK**  **LHSVPGLLGVQER**  **GPLNWYRNMRPNWRWALSAKDRKILMPALMVTAGKDVVLLPSMSKGMEEWIPQLR** |
| 35 | 3-oxo-5-β-steroid 4-dehydrogenase isoform 2 | *G. gallus* | 655 | **VPLSDGNSIPLLGLGTYADPQKTPK**  **IAIDTGYRHIDGAFVYYNEHEVGQAIR**  **REDIFYCGKLWNTCHPPELVRPTLEK**  **SIGVSNFNRRQLEMILNKPGLKHKPVSNQVECHPYFTQPKLLEFCRQHDIVIVGYSPLGTSRDETWVNVSSPPLLEDPVLNAIGKKYNKTAAQVALR**  **GVVVIPKSFNPQR**  **ENFQIFDFSLTEKEMKEIEALNKNVRYVELLMWRDHPEYPFNDEY** |
| 36 | α-Enolase | *G. gallus* | 93 | **GVPLYR**  **SCNCLLLKVNQIGSVTESLQACKLAQSNGWGVMVSHR**  **YNQLLR** |
| 37 | Phosphoglycolate phosphatase | *G. gallus* | 364 | **GEAALSGAPAALGR**  **TRVAYTEKLRRLGFPPAEPRHVFGSAFCAAR**  **AVLVGFDEHFSYAK**  **GPDCLLVGTNRDNRLPLEGGSAIPGTGCLVK**  **YIFDCVASEFDIDPAR** |
| 38 | Regucalcin | *G. gallus* | 1279 | **SSVKIECVGSDRYRLGESPVWDEKENSLLCVDITGR**  **KSGDYVITLGTR**  **WKEQLVTTIAQVDRDK**  **FNDGKVDPAGRYFAGTMAEEIRPAVLERRQGSLYTLCPDHSVVKHFDQVDISNGLDWSLDHK**  **SVYKLEKEESIPDGMCIDTEGKLWVACYDGGRVIRLDPETGKRIQTVKLPVDKTTSCCFGGKDYSEMYVTSASDGMDREWLSRQPQAGGVFK**  **GIPPYPFAG** |
| 40 | Hsp108 | *G. gallus* | 86 | **EVEEDEYKAFYK**  **SILFVPNSAPRGLFDEYGSKK** |
| 41 | Betaine--homocysteine S-methyltransferase | *G. gallus* | 316 | **VNEAACDIAR**  **QLDIFMKK**  **AGASIVGVNCHFDPDTVLETVKLMKEGLQAAK**  **AHLMSQPLAFHTPDCGKQGFIDLPEFPFGLEPR**  **WDVQKYARKAYDLGIRYIGGCCGFEPYHVRAIAEELAPERGFLPEASEK** |
| 42 | Prolyl-4-hydroxylase | *G. gallus* | 146 | **SVSDYEGKLDNFK**  **LITLEEEMTKYKPESDDLTADK**  **MDSTANEVEAVKIHSFPTLK**  **NVIDYNGER** |
| 43 | Protein disulfide-isomerase A3 precursor | *G. gallus* | 589 | **LAPEYEAAATRLKGIVPLVK**  **YGVSGYPTLKIFRDGEESGTYDGPRTADGIVSHLKKQAGPASVALSSVADFEKFIGDKDASVVGFFRDASGDAYSEFMK**  **FAHTSEEQLVQK**  **LANKFEDSTVKYTEDKITSAK**  **DLLVAYYDVDYEK** |
| 44 | α-Enolase | *G. gallus* | 62 | **NVNVVEQEKIDK**  **GVPLYR**  **IGAEVYHNLK**  **DGKYDLDFK** |
| 45 | Elongation factor 2 | *G. gallus* | 94 | **ETVSEESNVMCLSK**  **YLAEKYEWDVTEAR**  **EGVLCEENMR**  **GGGQIIPTAR**  **CLYACVLTAQPR** |
| 46 | Hsp108 | *G. gallus* | 56 | **EEASDYLELDTVK**  **TETVEEPVEEEEAK** |
| 47 | α-Enolase | *G. gallus* | 313 | **DATNVGDEGGFAPNILENK**  **VVIGMDVAASEFYRDGKYDLDFKSPDDPSR**  **SCNCLLLKVNQIGSVTESLQACKLAQSNGWGVMVSHR**  **YNQLLRIEEELGSKAR** |
| 48 | Apolipoprotein A-I | *G. gallus* | 59 | **LTPVAQELK**  **LTPVAEEAR**  **GIPQASEYQAK** |
| 49 | Annexin A6 | *G. gallus* | 71 | **GFGSDKDAILDLITSR**  **VEICQAYK**  **EIKDAIAGIGTDEK**  **DLEADVVGDTSGHFKK** |
| 50 | Glutathione S-transferase 2 | *G. gallus* | 546 | **VVTLGYWDIRGLAHAIRLLLEYTETPYQER**  **AGPAPDFDPSDWTNEKEK**  **LTQSNAILR**  **KHNMCGETEVEKQRVDVLENHLMDLR**  **LCYSPDFEKLKPAYLEQLPGK**  **LTFVDFLAYDVLDQQR**  **ISAYMR**  **APIFWYTALWNNK** |
| 51 | Hsp108 | *G. gallus* | 402 | **EEEAIQLDGLNASQIK**  **SEKFAFQAEVNR**  **LIINSLYKNKEIFLRELISNASDALDKIRLISLTDENALAGNEELTVK**  **NMLHVTDTGIGMTKEELIK**  **IGMTKEELIKSEFLNK** |
| 53 | Dihydropyrimidinase | *G. gallus* | 64 | **GCSLIEAFDKWK**  **AAVAGGTTMIIDFAIPQK** |
| 54 | Nucleoside diphosphate kinase | *G. gallus* | 492 | **GSAPPELREQTLVLVKPDAVQRRLVGDVIGRFER**  **GFKLVAMK**  **GLLDRHYQHLQQK**  **AMVGDTDSAQAAAGTIRGDLSMHVSRNVVHASDSVETALREIGFWFQR** |
| 55 | Alcohol dehydrogenase 6 | *G. gallus* | 130 | **IIAIDINKDK**  **EMGATECINPQDFK**  **TCKGTLAGGWKMR**  **LVASYLEKKFNSDLLITHTLPFAK** |
| 56 | Phosphoenolpyruvate carboxykinase | *G. gallus* | 134 | **LFHVNWFLR**  **FVWPGFGHNARVLAWIFGR**  **DTARPTPIGWVPK**  **GFWEEECR**  **EYYGENFGADLPRDVMAELEGLEER** |
| 57 | Fatty acid-binding protein | *G. gallus* | 461 | **YELQSHENFEPFMKALGLPDDQIQKGK**  **SISEIVQNGNKFKITVTTGSK**  **AKCIVNMEGNNKLVANLKGLKSVTELNGDTITHTMTK** |
| 58 | Phosphoglycerate kinase | *G. gallus* | 333 | **IVKDLMAK**  **KFVEVVGR**  **QIVWNGPVGVFEWDKFSK**  **ALMDKVVEVTGK**  **VSHVSTGGGASLELLEGK** |

^a^ Spot no. corresponds to the number of protein spots in Figure S1. Mascot scores are derived from ion scores as a non-probabilistic basis for ranking protein hits.
