# Supplementary material for: Probiotic (Enterococcus faecium) induced responses of the hepatic proteome improves metabolic efficiency of broiler chickens (Gallus gallus)
Source: BMC Genomics. 2016 Feb 1;17:89. doi: 10.1186/s12864-016-2371-5 (PMC4736614; doi:10.1186/s12864-016-2371-5)
Supplement: Additional file 3: Table S3. — Peptides identified from the liver of AA broiler chickens based on Mascot scores. (DOC 103 kb) [file 12864_2016_2371_MOESM3_ESM.doc]

**Table S3.** **The primer sequences used for qPCR analysis of the differentially expressed proteins of the liver of AA broiler chickens**

| Spot  no. | Symbol ID | Accession no. | Gene name | Primer sequence (5′→3′) | Product  size (bp) | *T*m  (°C) |
| --- | --- | --- | --- | --- | --- | --- |
| 24 | BHMT | gi|50755288 | Betaine-homocysteine S-methyltransferase 1 | GCTGCAAGGACAAAGCAGAG  AACTGGCTTGCCGGATTCTT | 155 | 62 |
| 12, 13 | CAT | gi|53127216 | CAT catalase | CCATCCTTCATCCATAGCCAGA  CCATCAGGAATACCACGATCAC | 140 | 62 |
| 33 | CTH | gi|118094764 | Cystathionase | TGCTGTTCTGTGTGTCCGTT  TGTGTCTCATGGCTGCTCTC | 95 | 62 |
| 32 | GOT1 | gi|45384348 | Aspartate aminotransferase | TCCAGTGAAGCACACGAACA  ATGGCAAGCCGTGAGTAAGA | 84 | 62 |
| 14 | FGB | gi|267844833 | Fibrinogen beta chain | CAGGCAATGCTCTGATGGAA  TACCACCATCCACCACCATC | 166 | 62 |
| 16, 18 | FGG | gi|45384500 | Fibrinogen gamma chain | GTCACCAGATGACACCACAGA  TGAACACGGCATAGTCAGCA | 143 | 62 |
| 42 | P4HB | gi|312283582 | Prolyl-4-hydroxylase | GGATTCCAGTGAAGTGGTTGTG  CTTGGCTAAGCTGGTACTTGGA | 152 | 62 |
| 43 | PDIA3 | gi|45383890 | Protein disulfide-isomerase A3 | CGAGATGGCGAAGAGTCAGG  AGCCACAGAACTGAGAGCCA | 111 | 62 |
| 29 | PDIA4 | gi|57530768 | Protein disulfide-isomerase A4 | AGGCCAGCCTGTTGACTATG  CAGAGTAGCTTCTGGTGGAGG | 106 | 62 |
| 48 | APOA1 | gi|45382961 | Apolipoprotein A-I | CAAGAACCTGGCGCCATACA  GCGTCATCTTCTCACGCAGG | 146 | 62 |
| Ref 1 | 28S | FM165415.2 | 28S rRNA | AGGTGCAGATCTTGGTGGTAG  CGCTTAGGACCAACTGACC | 112 | 62 |
| Ref 2 | ACTB | gi|211236 | β-Actin | GATGTGGATCAGCAAGCAGG  AAGCCATGCCAATCTCGTCT | 151 | 62 |
